# Predictors of Clinically Important Improvements in Motor Function and Daily Use of Affected Arm after a Botulinum Toxin A Injection in Patients with Chronic Stroke

**DOI:** 10.3390/toxins14010013

**Published:** 2021-12-23

**Authors:** Jen-Wen Hung, Wen-Chi Wu, Yi-Ju Chen, Ya-Ping Pong, Ku-Chou Chang

**Affiliations:** 1 Department of Rehabilitation, Chang Gung Memorial Hospital-Kaohsiung Medical Center, Kaohsiung 83301, Taiwan; wendy.wu224@gmail.com (W.-C.W.); anita61021@cgmh.org.tw (Y.-J.C.); yaping0707@gmail.com (Y.-P.P.); 2 School of Medicine, College of Medicine, Chang Gung University, Taoyuan 33302, Taiwan; kcchang@cgmh.org.tw; 3 Division of Cerebrovascular Diseases, Department of Neurology, Chang Gung Memorial Hospital, Kaohsiung 83301, Taiwan

**Keywords:** stroke, upper limb spasticity, motor function, botulinum toxin, predictors

## Abstract

Identifying patients who can gain minimal clinically important difference (MCID) in active motor function in the affected upper extremity (UE) after a botulinum toxin A (BoNT-A) injection for post-stroke spasticity is important. Eighty-eight participants received a BoNT-A injection in the affected UE. Two outcome measures, Fugl–Meyer Assessment Upper Extremity (FMA-UE) and Motor Activity Log (MAL), were assessed at pre-injection and after 24 rehabilitation sessions. We defined favorable response as an FMA-UE change score ≥5 or MAL change score ≥0.5.Statistical analysis revealed that the time since stroke less than 36 months (odds ratio (OR) = 4.902 (1.219–13.732); *p* = 0.023) was a significant predictor of gaining MCID in the FMA-UE. Medical Research Council scale -proximal UE (OR = 1.930 (1.004–3.710); *p* = 0.049) and post-injection duration (OR = 1.039 (1.006–1.074); *p* =0.021) were two significant predictors of MAL amount of use. The time since stroke less than 36 months (OR = 3.759 (1.149–12.292); *p* = 0.028), naivety to BoNT-A (OR = 3.322 (1.091–10.118); *p* = 0.035), and education years (OR = 1.282 (1.050–1.565); *p* = 0.015) were significant predictors of MAL quality of movement. The findings of our study can help optimize BoNT-A treatment planning.

## 1. Introduction

Spasticity after stroke is common. Approximately 43% and 38% of patients are found to have spasticity at 6 months [1] and 12 months [2] post-stroke, respectively. Spasticity significantly reduces the affected upper extremity (UE) motor function, which is required for daily life activities [3]. Increased daily arm use is important for patients with chronic stroke in order to maximize gains in their quality of life [4]. Therefore, treating spasticity to improve active function is an important issue in stroke UE rehabilitation training.

Botulinum toxin A (BoNT-A) is commonly used for treating focal spasticity [5]. Although the efficacy of BoNT-A in spasticity reduction is well established, the impact of active functional outcomes is controversial [6,7,8].The International Consensus Statement declared that motor function improved in some patients after BoNT-A injections; however, more studies are needed to elucidate the effects [9].

The controversial results [6,7,8] may be due to the fact that that not all patients with spasticity could benefit from active motor function after BoNT-A treatment. Many interacting factors may affect treatment outcomes. Thus, predictive models should be developed to identify responders and non-responders to interventions [10]. Despite the extensive use of BoNT-A for spasticity treatment, the patient cohort that might benefit the most in active function after BoNT-A treatment remains unclear. Detecting predictors may help to adapt treatment plans and more correctly stratify patients for a better outcome from BoNT-A treatment. In this study, we selected two active functional outcome measures, the Fugl–Meyer Assessment upper extremity (FMA-UE) [11] and Motor Activity Log (MAL) [12], in reference to the International Classification of Functioning, Disability, and Health (ICF) framework published by the World Health Organization [13].The FMA-UE represents the body function domain, while MAL represents activity and participation domains.

Labeling the responders to each intervention is an important issue. Efficacy is defined as the improvement in some determined outcome measures in response to a specific treatment. Statistically significant changes are usually used to prove efficacy. However, for each outcome measure, it is important to know if the change is meaningful, rather than statistically significant. A statistically significant difference does not necessarily convert to clinically meaningful improvements [14]. In some studies with large sample sizes, small effects could be statistically significant, but these effects may have no clinical relevance. The idea of minimal clinically important difference (MCID) was proposed by Jaeschke et al. in 1989 [15].MCID defines a threshold that is considered to be an important change; this measure prevents the problem of mere statistical significance and provides valuable information for clinical practice.

This study aimed to explore the predictors of clinically important changes in active motor function and daily use of the affected UE after a BoNT-A injection for post-stroke UE spasticity.

## 2. Results

Eighty-eight participants with chronic stroke that received a BoNT-A injection in the affected UE and 24 sessions of rehabilitation training within 4 months post injection were evaluated. A total of 62 men and 26 women, with a mean age of 49.32 ± 10.95 years, were enrolled.Fifty-one patients (57.95%) had left hemiplegia, and 53patients (60.2%) had cerebral infarction. The time since stroke onset to BoNT-A injection was 33.23 ± 22.44 months. Forty-eight (54.55%) patients were naïve to BoNT-A injection. The baseline demographic and clinical characteristics of the participants are shown in Table 1.

The mean total injection dose was 327U (range 100–400 U). The injected muscles and doses were the pectoralis major (62.86 ± 16.84 U in 14 (15.91%) patients); the biceps brachii (53.33 ± 25.17 U in three (3.41%) patients); the brachialis (63.69 ± 24.64 U in 65 (73.86%) patients); the brachioradialis (46.00 ± 23.75 U in 50 (56.82%) patients); the triceps brachii (43.33 ± 16.14 U in 12 (13.64%) patients); the pronator teres (43.72 ± 13.90 U in 74 (84.09%) patients; the pronator quaratus (32.13 ± 10.43 U in 40 (45.45%) patients); the flexor carpi radialis (48.01 ± 16.21 U in 68 (77.27%) patients); the flexor carpi ulnaris (29.52 ± 10.86 U in 52 (59.09%) patients); the flexor digitorum profundus (34.46 ± 17.12 U in 28 (31.82%) patients); the flexor digitorum superficialis (85.48 ± 33.46 U in 84 (95.45%) patients); the lumbricallis (19.13 ± 7.64 U in 23 (26.14%) patients); the pollicis adductor(10.96 ± 3.47 U in 26 (29.55%) patients); and the flexor pollicis longus (27.29 ± 7.17 U in 72 (81.82%) patients).

No serious adverse events were observed. Three (3.41%) patients reported muscular weakness after injection: grasping, forearm pronation, and elbow flexion, respectively. All patients had mild symptoms for less than 1 month, and no intervention was indicated. No participants withdrew due to AEs.The outcome measures were assessed at 76.18 ± 17.18 days post injection. Based on the information collected for the 88 patients, all outcomes showed statistically significant improvements after the intervention (*p* < 0.01) (Table 2). These results indicated that patients had better motor function as well as greater and better use of the affected UE after a BoNT-A injection and rehabilitation training.

Twenty-five (28.41%), 45 (51.14%), and 37 (42.45%) participants reached the MCID for FMA-UE, MAL amount of use (AOU), and MAL quality of movement (QOM), respectively. The results of χ2 and independent-sample t tests revealed three predictors: the time since stroke less than 36 months, Wolf Motor Function Test (WMFT) quality score, and Medical Research Council scale (MRC)-distal UE score for the multivariate logistic regression analysis of the FMA model. Five predictors: naïve to BoNT-A, education years, post-injection duration, MRC proximal UE, and proprioception scores were selected for the MAL AOU model. Six predictors such as the time since stroke less than 36 months, education years, naïve to BoNT-A, post-injection duration, FMA-UE proximal, and proprioception scores were selected for the MAL QOM model (Table 3).

Table 4 summarizes the results of multivariate logistic regression analyses. The time since stroke less than 36 months (odds ratio (OR) = 4.902 (1.219–13.732); *p* = 0.023) was a significant predictor of gaining MCID in the FMA-UE. MRC-proximal UE (OR = 1.930 (1.004–3.710); *p* = 0.049) and post-injection duration (OR = 1.039 (1.006–1.074); *p* = 0.021) were the two significant predictors of MAL AOU. The time since stroke less than 36 months (OR = 3.759 (1.149–12.292); *p* = 0.028), naivety to BoNT-A (OR = 3.322 (1.091–10.118); *p* = 0.035), and education years (OR = 1.282 (1.050–1.565); *p* = 0.015) were significant predictors of MAL QOM.

All variance inflation factor (VIF) values of the predictors were <10 (range: 1.02–1.34), indicating the presence of weak multicollinearity. The *p* values of all Hosmer–Lemeshow tests were >0.05, which represented the consistency of the model’s predictions with the expectations of the model itself.

## 3. Discussion

To the best of our knowledge, this is the first study to examine clinically significant improvements in motor function and daily use of the affected UE after BoNT-A injections. Patients with the time since stroke less than 36 months had a greater chance of achieving clinically significant improvements in FMA-UE. Patients with a longer post-injection duration and/or greater proximal UE muscle strength could use their affected UEs more frequently in activity of daily living (ADL). Patients with a time since stroke less than 36 months, naïve to BoNT-A injection, and/or higher education level had a greater possibility of achieving MCID in MAL QOM.

Our results support the importance of the time since stroke in active function gained after a BoNT-A treatment. An investigation revealed that BoNT-A could be more helpful in subacute patients than in chronic patients for decreasing spasticity, contracture, and improving function [16]. Furthermore, an international consensus statement recommended early BoNT-A injection for active functional improvement [9]. By recruiting patients with chronic stroke, we demonstrated that injecting BoNT-A in the affected UE earlier than 3 years post-stroke may be more beneficial for active motor function gains.

The post-injection duration was relevant to the improvement in the frequency of UE use in ADL. As all patients received the same dosage of training, the post-injection duration represented the time patients could self-practice at home. We believe that the amount of practice in ADL is an important factor for the increase in the frequency of use of the affected UE in daily life.

One of the probable reasons for the lack of active effect from a BoNT-A injection is the greater role of weakness than spasticity on functional disability. Thus, patients with better underlying strength may benefit more from BoNT-A injections [7]. We found that participants with greater muscle strength of proximal UE had a higher probability of achieving MCID in the MAL AOU. Reaching is an essential element of many daily living activities. The reaching performance of the affected UE after stroke is reported to markedly depend on UE muscle power [17]. The act of reaching out requires proximal UE muscle power to take the weight of the UE or stabilize it in space. Patients with greater muscle strength of the proximal UE at baseline could have better reaching ability after BoNT-A injection and would use the affected UE more in ADL than those with less muscle strength of proximal UE.

Using the UE to perform ADL requires fine (e.g., grasping) and coarse (e.g., reaching) motor abilities. Several studies have indicated that manual dexterous function significantly affects patient’s ADL performance [18,19]. However, our study did not reveal such results. In our study, neither the MRC-distal UE nor the FMA-UE distal score could predict affected UE use in ADL, as shown in MAL. One possible reason for this result is the recruitment of moderate to severe cases and the generally poor manual dexterity in our patients.

We found that patients with higher education had a higher chance of gaining MCID of MAL QOM. No previous studies have reported that years of education could be a predictor of UE functional improvement after interventions. We suspected that the higher education level may be related to higher learning ability to improve the quality of affected UE use in ADL. Further studies should be conducted to clarify the influence of the years of education.

Spasticity in the UE might interfere with the performance of UE movements. Several studies have revealed that less spasticity predicts greater improvements in UE rehabilitation training [20,21,22]. In our study, we did not find the MAS of UE at baseline as a negative predictor of active function gain. Such findings may be due to all patients receiving BoNT-A treatment and the decrease in the spasticity effect.

The selection bias for recruiting patients that are naïve or non-naïve to BoNT-A treatment is concerning, as non-naïve patients maybe more tolerable to BoNT-A treatment, which might also be more effective in this cohort [23]. However, we found that patients who were naïve to BoNT-A had a higher chance of gaining the MCID of MAL QOM. One explanation is that the non-naïve to BoNT-A patients had better MAL QOM than naïve patients (1.07 ± 0.85 vs. 0.75 ± 0.54), and a ceiling effect occurred for non-naïve patients. Despite the accumulated effects after repeated injections [24,25,26], patients naïve to BoNT-A may have more potential to improve their QOM than non-naïve patients for one BoNT-A injection. After the first BoNT-A injection, training the functional use of the affected UE in ADL is strongly suggested.

BoNT-A injection programs, such as concentration, dosage, number of injected muscles, injectors, and co-interventions, may influence the outcome of BoNT-A treatment. We standardized some BoNT-A injection procedures, such as the use of same concentration (50 U BoNT-A per 1 mL), performance of echo guidance for injection localization, and injection administered by only two physiatrists, who had similar injection experience and principle. We found that the total injection dosage was not a predictor of active function gain. There were also no differences between the two injectors in any of the outcome measures (data not shown). However, it is difficult to standardize other factors. The muscles selected for the BoNT-A injection and dose per muscle were individualized according to patterns and severity of spasticity. We could not identify the effects of the number of injected muscles or the dosage of each injected muscle. In the future, a large study with subgroups of standardized BoNT-A injection programs according to patients’ conditions is needed to assess the effects of the diversity of BoNT-A injection programs and to build a standardized program for BoNT-A treatment for post-stroke UE spasticity.

Safety is an important issue in BoNT-A treatment. The Adult Spasticity International Registry (ASPIRE) study is a large real-world, 2-year follow-up study on onabotulinumtoxin A utilization for spasticity. In the UE results, 15 treatment-related adverse events (AEs) were found in 14 patients (2.9%). The most common AE was muscular weakness (1.4%) [23]. In our study, similar findings were observed in three patients (3.41%) who reported muscular weakness after injection: one for grasping, one for forearm pronation, and one for elbow flexion. All patients had mild symptoms lasting less than 1 month. Intervention was not indicated and no patient withdrew from the study due to AE. No other AEs were reported. The patient who had post-injection grasping weakness achieved MCID of FMA and MAL AOU and QOM after the intervention. The patient who had post-injection forearm pronation weakness achieved MCID of MAL AOU and QOM after the intervention. The patient who had post-injection elbow flexor weakness achieved MCID of MAL AOU after the intervention. Post-injection weakness might not be an absolute negative factor for active function gains. The number of AEs in our study was small; therefore, we did not perform further analyses. A large study is needed to assess the impact of BoNT-A-related AEs on functional gains.

### Study Limitations

Our results may provide clinicians with simple and valuable tools to identify patients who could have clinically important gains in motor function and UE use after BoNT-A injection. However, in the interpretation of our results, caution should be exercised owing to the limitations of this study. First, this was a retrospective, secondary data analysis study that had potential confounding factors. Second, we had a small sample size. Considering the limited case number, only candidate predictors with a significance level of *p* < 0.05 identified by χ2 and independent-sample *t*-tests were entered into the multivariate logistic regression models. Some potential factors may be excluded under such strict filters. In addition, we included only the potential predictors of baseline descriptive characteristics and clinical assessments. Other potential factors that might affect treatment outcomes, such as psychosocial function, occupation, contextual variables, or kinematic parameters, need further investigation to improve the model. Third, the homogeneity of patients in some variables, such as cognition function and proprioception deficits, may have resulted in the non-significance of these variables in predicting important changes in outcome measures. Fourth, because we only investigated the predictors of functional gain after one injection, no information on the predictors of repeated injection outcomes is presented. Fifth, our endpoints were only up to 4 months after a single injection; however, long-term follow-up might be needed to assess the maintenance effect. Sixth, combining a rehabilitation program after BoNT-A injection was suggested as an approach to optimize spasticity treatment outcomes [5]. There is no agreement on the most effective approach [27]. All patients received 24 sessions of rehabilitation training; however, the training programs varied. We did not consider the effects of the different adjuvant therapies as each training program only included a small number of participants.

## 4. Conclusions

The time since stroke less than 36 months, longer post-injection duration, greater muscle strength of the proximal UE, more education years, and naïve to BoNT-A treatment are valuable in predicting favorable outcomes in motor function and the use of the affected UE following stroke spasticity treatment with BoNT-A. Both predictors and the outcome measures are clinically relevant and can be easily conducted, which make our results valuable for clinical applications. The findings of our study can help optimize BoNT-A treatment planning. Further studies with a more comprehensive set of factors, a larger number of patients, repeated injections, and long-term follow-up are recommended for improving the predictive models.

## 5. Materials and Methods

### 5.1. Patients and Experimental Setup

This was a retrospective, secondary data analysis. For this study, we originally enrolled 88 participants from our previous studies that aimed to determine the effects of BoNT-A injection combined with rehabilitation training for post-stroke spasticity. The requirement for informed consent was waived owing to the retrospective design of the study.

The inclusion criteria were as follows: (1) clinical and imaging diagnosis of a first or recurrent unilateral stroke of ≥6 months; (2) UE spasticity (at least one UE muscle with a MAS ≥ 1+; (3) initial motor part of the FMA-UE score ranging from 13 to 56, indicating moderate to severe movement impairment; (4) Mini Mental State Exam score > 20, indicating no serious cognitive impairment; and (5) age ≥ 18 years. The exclusion criteria were as follows: (1) bilateral hemispheric or cerebellar lesions; (2) severe aphasia; (3) significant visual field deficits; (4) treatment with BoNT-A ≤ 4 months before recruitment; or (5) history of orthopedic or other neurologic diseases or medical conditions that would prevent adherence to the rehabilitation protocol [28].

### 5.2. Procedure

After a baseline assessment, participants received a BoNT-A injection for UE spasticity by one of two senior rehabilitation physicians. Botox brand BoNT-A purified neurotoxin complex (Allergan, an AbbVie Company, Irvine, CA, USA) was used. We added 2 mL normal saline into one vial of BoNT-A (100 U) to achieve a BoNT-A concentration of 50 U per 1 mL and a maximum dose of 400 units [29,30]. We used echo guidance to localize the targeted muscle. Muscles selected for BoNT-A injection and dose per muscle were individualized according to patterns and severity of spasticity. Within 1 week after the injection, all participants received 24 rehabilitation training sessions by well-trained occupational therapists. There were 2–4 training sessions per week for 6–12 weeks. Each training session included 45–60 min of robot-assisted training, mirror therapy, or traditional occupational therapy, followed by 30 min of functional training; the raters were blinded to participants’ treatment allocation. The following assessments were extracted before BoNT-A injection (T0) and after 24 sessions of rehabilitation therapy (T1). BoNT-A related AEs during the intervention period were also recorded.

### 5.3. Outcome Measures

#### 5.3.1. FMA-UE

FMA-UE evaluates UE motor impairments of stroke patients. The score ranges from 0 to 66, with higher scores indicating fewer motor deficits. FMA-UE has good reliability, validity, and responsiveness in patients with stroke [11,31,32]. We defined the MCID of the FMA-UE as a change score of ≥5 points based on previous reports [33,34].

#### 5.3.2. MAL

MAL is a semi-structured interview questionnaire in which patients rate AOU and QOM while using their affected UE to accomplish 30 daily activities. The score ranges from 0 (never using the affected UE for the activity) to 5 (ability to use the affected UE for that task as good as before stroke), with higher scores indicating better performance. MAL has good reliability and concurrent validity [12]. Changes in MAL scores could reflect improvements in both participation and functional independence [35,36].The values of MCID were estimated to be approximately 10% of the range of the scale for chronic patients [37].Thus, we defined the MCID in MAL AOU and MAL QOM after BoNT-A injection as an improvement of ≥0.5 [12,38].

### 5.4. Potential Predictors

We selected potential predictors according to previous studies of upper limb recovery in stroke patients [30,39]. The potential predictors included the demographic (age, sex, education, and cognition level), clinical records (time since stroke, side of lesion, type of stroke, lesion location, naïve to BoNT-A or not, post injection duration, and injection dose), and clinical assessment scores (MAS, MRC, joint proprioception score, FMA-UE, WMFT, MAL).

#### 5.4.1. MAS

We used MAS, which has shown good reliability and validity, to assess the severity of spasticity [40,41]. For statistical analysis, we record a 1+ score as 1.5. In addition, we estimated mean MAS scores of wrist flexors/extensors and finger flexors/extensors in each participant as the MAS of the distal UE and the mean of MAS scores of shoulder flexors/extensors, abductors/adductors, internal/external rotators, elbow flexor/extensors, and forearm pronator/supinator as the MAS of the proximal UE for further analysis.

#### 5.4.2. MRC

We used the MRC scale to measure limb muscle power. The MRC scale is a reliable measurement that ranges from 0 (no contraction) to 5 (normal power) [42,43]. We estimated the mean MRC score in the shoulder flexors/abductors, elbow flexors/extensors as MRC proximal, mean MRC score in the wrist flexors/extensors, and finger flexors/extensors as MRC distal.

#### 5.4.3. Joint Proprioception Sensation

The Revised Nottingham Sensory Assessment (rNSA) [44] is a reliable scale for assessing sensory function in stroke patients. Proprioception subscales of the rNSA were extracted. The affected UE was supported and moved by the assessor in various directions at one joint at a time. The patient was asked to mirror the change in movement with the unaffected UE. A scoring of 0 indicated no appreciation of movement taking place; 1, the patient could appreciate and mirror the direction of the test movement taking place each time but is inaccurate in the new position; and 2, accurately mirrors the test movement to within 10” of the new test position. We estimated the mean score of the affected shoulder, elbow, wrist, and fingers for further analysis.

#### 5.4.4. WMFT

The WMFT is a quantitative measure of UE motor ability by using timed and functional tasks [45]. The WMFT includes 17 tasks. Performances were timed and rated using a 6-point ordinal scale. WMFT has good interrater reliability and criterion validity for patients with hemiparesis [46].

#### 5.4.5. Time since Stroke Onset to BoNT-A Injection

Due to the fact that, in our study, the mean time since stroke onset to BoNT-A injection was 33 months, we divided the patients to two groups as with the time since stroke onset to BoNT-A injection less than 36 months vs. not less than 36 months for further analysis.

### 5.5. Data Analysis

Paired *t*-tests were used to compare FMA and MAL data between pre-intervention and post-intervention assessments. A patient with a changed score that reached the MCID for the FMA (≥5) or the MAL (≥0.5) was coded as 1 (responder), and a patient who did not reach the MCID was coded as 0 (non-responder).

We used χ2 and independent sample *t*-tests to compare the participants’ baseline characteristics between responders and non-responders to identify the potential predictors of achieving MCID in FMA-UE and MAL scores.

Considering the limited case number, the candidate predictors with a significance level of *p* < 0.05, identified by χ2 and independent-sample *t*-tests, were entered into the multivariate logistic regression models with an enter procedure. Predictive equations and odds ratios of the significant predictors were generated from the analyses, with a significance level of *p* < 0.05. In order to ensure the quality of the models, VIF and Hosmer–Lemeshow goodness-of-fit tests were used. A VIF value of <10 indicates the absence of multicollinearity. A Hosmer–Lemeshow test *p*-value > 0.05 indicated the consistency of the model’s predictions with the expectations of the model itself [47].

All tests were executed using the SPSS software version 25 (International Business Machines Corp., Armonk, NY, USA) at a significance level of α = 0.05.

## Figures and Tables

**Table 1:** Demographics for the 88 participants.

Characteristics	Value
Age (years)	49.32 ± 10.95
Sex (Male/Female)	62 (70.5)/26 (29.5)
Education years	12.09 ± 3.51
Side of Hemiplegia (Rt/Lt)	37 (42.0)/51 (58.0)
Nature (Hemorrhage/Infarction)	35 (39.8)/53 (60.2)
Lesion (Cortical/Subcortical/Brainstem)	29 (33.0)/58 (65.9)/1 (/1.1)
Naïve to BoNT-A (Yes/No)	48 (54.55)/40 (45.45)
Total injection dose (U)	326.70 ± 95.66

NOTE. Values are mean ± SD or N (%). BoNT-A, Botulinum toxin A.

**Table 2:** Comparison of pre and post-intervention assessments within the 88 participants.

Outcome	Mean ± SD	*p*
FMA_UE		
Baseline	30.11 ± 8.25	<0.01
Post-treatment	33.20 ± 8.30	
MAL AOU		
Baseline	1.27 ± 0.72	<0.01
Post-treatment	1.82 ± 0.84	
MAL QOM		
Baseline	0.90 ± 0.70	<0.01
Post-treatment	1.37 ± 0.81	

NOTE. Values are mean ± SD. FMA_UE, upper limb subtest of the Fugl–Meyer assessment; MAL AOU, Motor Activity Log Amount of use score; MAL QOM, Motor Activity Log Quality of movement score.

**Table 3:** Univariate analysis comparing patients with or without clinically important improvements after intervention in the FMA-UE and MAL.

Candidate Predictor	FMA-UE			MAL AOU			MAL QOM		
	Change ≥ 5	Change < 5	*p*	Change ≥ 0.5	Change < 0.5	*p*	Change ≥ 0.5	Change < 0.5	*p*
	*n* = 25	*n* = 63		*n* = 45	*n* = 43		*n* = 37	*n* = 51	
**General Information**									
Age (years)	46.19 ± 13.67	50.56 ± 9.37	0.159	48.59 ± 11.47	50.09 ± 10.31	0.527	47.70 ± 11.36	50.50 ± 10.48	0.249
Sex (male/female)	17/8	45/18	0.751	29/16	33/10	0.206	25/12	37/14	0.613
Education years	11.52 ± 3.90	12.32 ± 3.31	0.381	12.82 ± 3.41	11.33 ± 3.44	0.046 ^†^	13.43 ± 2.95	11.12 ± 3.55	0.001 ^†^
Time since stroke (<36 months/≧36 months)	21/4	34/29	0.014 ^†^	31/14	24/19	0.205	29/8	26/25	0.009 ^†^
Lesion Side (left/right)	11/14	26/37	0.815	21/24	16/27	0.369	20/17	17/34	0.052
Nature (hemorrhage/infarction)	8/17	27/36	0.348	18/27	17/26	0.964	16/21	19/32	0.571
Naïve to BoNT-A (Yes/No)	14/11	34/29	0.863	30/15	18/25	0.019 ^†^	28/9	20/31	0.001 ^†^
Post-injection days	75.88 ± 16.97	76.30 ± 17.12	0.918	81.87 ± 17.67	70.41 ± 13.53	0.001 ^†^	82.24 ± 19.07	71.78 ± 13.91	0.007 ^†^
Injection dose (U)	298.00 ± 88.14	338.10 ± 96.15	0.071	310.11 ± 84.82	344.07 ± 103.01	0.100	313.38 ± 82.69	336.37 ± 102.99	0.255
**Clinical Assessment at Baseline**									
MMSE	27.52 ± 2.35	26.92 ± 2.50	0.303	26.98 ± 2.60	27.21 ± 2.33	0.665	27.41 ± 2.64	26.86 ± 2.33	0.326
FMA-UE									
Proximal	26.76 ± 6.10	26.10 ± 6.42	0.657	27.49 ± 6.19	25.02 ± 6.25	0.070	28.43 ± 5.59	24.73 ± 6.40	0.005 ^†^
Distal	4.04 ± 2.54	3.84 ± 3.13	0.762	4.20 ± 2.93	3.58 ± 3.00	0.336	3.95 ± 2.58	3.86 ± 3.24	0.895
Proprioception score	8.36 ± 3.87	7.62 ± 3.38	0.415	8.82 ± 3.26	6.79 ± 3.53	0.007 ^†^	8.97 ± 3.14	7.00 ± 3.59	0.008^†^
MAS									
Proximal UE	7.66 ± 4.30	8.91 ± 3.31	0.206	8.51 ± 4.05	8.60 ± 3.22	0.906	8.84 ± 3.60	8.35 ± 3.70	0.544
Distal UE	3.18 ± 1.83	3.87 ± 1.74	0.115	3.63 ± 1.81	3.71 ± 1.77	0.843	3.89 ± 1.80	3.51 ± 1.76	0.323
MRC									
Proximal UE	13.72 ± 3.41	12.90 ± 3.17	0.317	13.89 ± 3.33	12.35 ± 2.99	0.026 ^†^	13.86 ± 3.14	12.61 ± 3.25	0.075
Distal UE	9.32 ± 3.40	7.38 ± 2.92	0.018 ^†^	8.09 ± 2.98	7.77 ± 3.38	0.642	7.68 ± 2.66	8.12 ± 3.51	0.508
WMFT									
Time (mean)	8.77 ± 4.19	10.87 ± 8.72	0.136	9.52 ± 4.94	11.06 ± 9.84	0.366	9.54 ± 4.40	10.80 ± 9.46	0.409
Quality (mean)	2.51 ± 0.40	2.25 ± 0.47	0.016 ^†^	2.39 ± 0.46	2.25 ± 0.47	0.161	2.42 ± 0.47	2.26 ± 0.46	0.118
MAL									
AOU (mean)	1.29 ± 0.62	1.26 ± 0.75	0.846	1.28 ± 0.65	1.27 ± 0.78	0.935	1.26 ± 0.61	1.28 ± 0.79	0.859
QOM (mean)	0.88 ± 0.63	0.91 ± 0.73	0.838	0.88 ± 0.57	0.92 ± 0.82	0.780	0.85 ± 0.51	0.93 ± 0.81	0.576

FMA-UE, upper limb subtest of the Fugl–Meyer assessment; MAL, Motor Activity Log; AOU, amount of use; QOM, quality of movement; BoNT-A, Botulinum toxin A; MMSE, Mini-Mental Status Examination; MAS, Modified Ashworth Scale; UE, upper extremity; MRC, Medical Research Council scale; WMFT, Wolf Motor Function Test; ^†^ indicates that the predictors were selected into the multivariate logistic regression analyses (*p* < 0.05).

**Table 4:** Multivariate logistic regression analysis for the predictors of clinically important changes in FMA-UE and MAL.

Predictor	FMA-UE			MAL AOU			MAL QOM		
	β	*p*	OR (95% CI)	β	*p*	OR (95% CI)	β	*p*	OR (95% CI)
Constant	−4.594	0.002		−7.225	<0.001		−9.921	<0.001	
Time since stroke less than 36 months	1.409	0.023 ^†^	4.092 (1.219–13.732)				1.612	0.0012^†^	5.013 (1.420–17.699)
Education year				0.099	0.199	1.104 (0.949–1.284)	0.248	0.015^†^	1.282 (1.050–1.565)
Naïve to BoNT-A				0.605	0.229	1.831 (0.683–4.910)	1.201	0.035^†^	3.322 (1.091–10.118)
Post-injection duration				0.039	0.021^†^	1.039 (1.006–1.074)	0.026	0.131	1.026 (0.992–1.061)
MRC proximal UE				0.657	0.049^†^	1.930 (1.004–3.710)			
MRC distal UE	0.567	0.135	1.762 (0.839–3.704)						
WMFT quality	0.633	0.333	1.883 (0.523–6.786)						
FMA-UE proximal							0.091	0.054	1.096 (0.999–1.202)
Proprioception				0.087	0.228	1.091 (0.947–1.257)	0.078	0.368	1.081 (0.913–1.280)

FMA-UE, upper limb subtest of the Fugl–Meyer assessment; MAL, Motor Activity Log; AOU, amount of use; QOM, quality of movement; BoNT-A, Botulinum toxin A; MRC, Medical Research Council scale; UE, upper extremity; WMFT, Wolf Motor Function Test; ^†^ indicates *p* < 0.05.

## Data Availability

The data presented in this study are available upon request from the corresponding author. The data are not publicly available due to ethical issues.
